# A Novel Framework for the Identification of Reference DNA Methylation Libraries for Reference-Based Deconvolution of Cellular Mixtures

**DOI:** 10.3389/fbinf.2022.835591

**Published:** 2022-03-21

**Authors:** Shelby Bell-Glenn, Jeffrey A. Thompson, Lucas A. Salas, Devin C. Koestler

**Affiliations:** ^1^ Department of Biostatistics and Data Science, University of Kansas Medical Center, Kansas City, KS, United States; ^2^ Department of Epidemiology, Geisel School of Medicine, Dartmouth College, Hanover, NH, United States

**Keywords:** reference-based deconvolution, IDOL, cell heterogeneity, cell proportion estimation, DNA methylation, EWAS

## Abstract

Reference-based deconvolution methods use reference libraries of cell-specific DNA methylation (DNAm) measurements as a means toward deconvoluting cell proportions in heterogeneous biospecimens (e.g., whole-blood). As the accuracy of such methods depends highly on the CpG loci comprising the reference library, recent research efforts have focused on the selection of libraries to optimize deconvolution accuracy. While existing approaches for library selection work extremely well, the best performing approaches require a training data set consisting of both DNAm profiles over a heterogeneous cell population and gold-standard measurements of cell composition (e.g., flow cytometry) in the same samples. Here, we present a framework for reference library selection without a training dataset (RESET) and benchmark it against the Legacy method (minfi:pickCompProbes), where libraries are constructed based on a pre-specified number of cell-specific differentially methylated loci (DML). RESET uses a modified version of the Dispersion Separability Criteria (DSC) for comparing different libraries and has four main steps: 1) identify a candidate set of cell-specific DMLs, 2) randomly sample DMLs from the candidate set, 3) compute the Modified DSC of the selected DMLs, and 4) update the selection probabilities of DMLs based on their contribution to the Modified DSC. Steps 2–4 are repeated many times and the library with the largest Modified DSC is selected for subsequent reference-based deconvolution. We evaluated RESET using several publicly available datasets consisting of whole-blood DNAm measurements with corresponding measurements of cell composition. We computed the RMSE and *R* ^2^ between the predicted cell proportions and their measured values. RESET outperformed the Legacy approach in selecting libraries that improve the accuracy of deconvolution estimates. Additionally, reference libraries constructed using RESET resulted in cellular composition estimates that explained more variation in DNAm as compared to the Legacy approach when evaluated in the context of epigenome-wide association studies (EWAS) of several publicly available data sets. This finding has implications for the statistical power of EWAS. RESET combats potential challenges associated with existing approaches for reference library assembly and thus, may serve as a viable strategy for library construction in the absence of a training data set.

## Introduction

Epigenome-wide association studies (EWAS) explore epigenetic variation, specifically in DNA methylation (DNAm). These studies provide insight into how environmental factors can influence disease, as well as indirectly inform potential novel therapeutics to treat human diseases (Rakyan et al., 2011; Michels et al., 2013; Flanagan, 2015). A well-recognized challenge in the statistical analysis and interpretation of EWAS, particularly EWAS that involve DNAm profiling of heterogeneous tissue types (e.g., whole blood, peripheral blood mononuclear cells, etc.), arises from the cell specificity of DNA methylation. It has been well documented that DNAm analyses of heterogeneous cell populations are prone to a decreased statistical power for detecting CpG-specific methylation effects or, worse, confounding and misleading results (Reinius et al., 2012; Jaffe and Irizarry, 2014; Liang and Cookson, 2014; Houseman et al., 2015). One obvious way to deal with the potential for confounding due to cellular heterogeneity is to adjust downstream statistical models for cellular composition (e.g., by adding terms to statistical models that reflect the proportion of each cell type within the heterogeneous sample used for methylation profiling). When available, cell composition is measured using complete blood cell counts (CBC) with differential or flow cytometry in the same biospecimens used for DNA methylation profiling (Houseman et al., 2015). However, these measurements are not routinely collected for most EWAS due to added cost to the study and technical limitations of these approaches (e.g., both CBC and flow cytometry require fresh samples and are not feasible for stored specimens).

To address these issues, methods for DNAm-based cell mixture deconvolution (CMD) have been developed. CMD relies on the assumption that methylation signatures for a heterogeneous sample can be thought of as a weighted mixture of the unique methylation signature of each cell type making up the sample (Houseman et al., 2012; Newman et al., 2015; Houseman et al., 2016; Koestler et al., 2016; Teschendorff et al., 2017; Titus et al., 2017; Decamps et al., 2020; Scherer et al., 2020). Thus, CMD makes use of the cell-specificity of DNAm to estimate the cell proportions in samples with heterogeneous cell composition. CMD includes both reference-free and reference-based approaches. Reference-free deconvolution obtains cell type-specific proportions when DNAm profiles in purified cell types are not available (Houseman et al., 2016; Decamps et al., 2020; Scherer et al., 2020). While broadly applicable across a range of different tissue types, a major limitation of reference-free deconvolution methods is that they are incapable of resolving the specific identity of the individual cell types for which deconvolution estimates are obtained. For this reason and because the identities of individual cell types are often of interest themselves (Koestler et al., 2017; Wiencke et al., 2017; Grieshober et al., 2018; Grieshober et al., 2021), reference-based deconvolution methods are often preferred over reference-free approaches when cell-specific methylation signatures are available for the cell types expected to be present in the tissue source being used for DNAm assessment. Reference-based methods use reference signatures of DNAm for the underlying cell types that make up a sample (reference libraries) (Houseman et al., 2012; Newman et al., 2015; Koestler et al., 2016; Teschendorff et al., 2017). Several reference-based methods for deconvoluting DNAm data have been proposed in recent years, including support vector regression (CIBERSORT), robust partial correlation (EpiDish), and constrained projection/quadratic programming (Houseman et al., 2012; Newman et al., 2015; Teschendorff et al., 2017). While the specific statistical method used for CMD has some impact on the accuracy of cell proportion estimates, the major factor underlying the performance of these methods is the reference library, or collection of cell-specific differentially methylated loci (DML), used as the basis for deconvolution (Koestler et al., 2016; Teschendorff et al., 2017; Titus et al., 2017). Reference libraries that result in accurate deconvolution estimates are comprised of cell-specific methylation biomarkers that exhibit distinct patterns of methylation across cell types.

To date, there have been several approaches for constructing reference libraries for DNAm-based CMD. The first approach assembles libraries using the $J^{*}\ll$ *J* CpGs that have the largest F-statistic computed from fitting a series ANOVA models independently to each CpG based on DNA methylation data of purified isolated cell types (Houseman et al., 2012; Koestler et al., 2016). For a given CpG, the ANOVA-based approach tests the null hypothesis that the mean beta value is the same across all *K* cell types versus the alternative hypothesis that the mean beta differs between at least two of the *K* cell types. Reference libraries assembled using this approach are limited in that they tend to be comprised of CpGs that distinguish some cell types well (e.g., neutrophils and lymphocyte subtypes, in the context of white blood cell types) but not cell types that share a common lineage (e.g., CD4T and CD8T) (Koestler et al., 2016). Improvements were made to the ANOVA approach by instead using the top hypermethylated and hypomethylated CpGs for each cell type, hereafter referred to as the Legacy approach. When using the Legacy approach, CpGs are selected by their t-statistic, computed from a series of two-sample t-tests fit independently to each CpG that test whether the mean beta value of each cell type is the same as the mean beta value across the *K*-1 remaining cell types. CpGs are rank-ordered based on their t-statistic, and those with the largest and smallest statistics are used to assemble a reference library for CMD (Aryee et al., 2014; Jaffe and Irizarry, 2014; Koestler et al., 2016). The Legacy approach is implemented in the Bioconductor package minfi using the function pickCompProbes (Aryee et al., 2014). While the Legacy method is an improvement over the ANOVA approach (Koestler et al., 2016), it is limited because it requires users to make arbitrary decisions about the number of hypermethylated and hypomethylated CpGs for each cell type used in assembling reference libraries. Further, the assumption of an equal number of DMLs for each cell type in the creation of reference libraries may hinder the accuracy of CMD-based estimates of cell proportions. Yet another approach, proposed by Koestler et al., called IDOL, iteratively searches CpGs in the construction of reference libraries that maximize the prediction accuracy of CMD-based estimates of cell proportions (Koestler et al., 2016). IDOL improves upon both the ANOVA and Legacy method in prediction performance but requires a training dataset consisting of whole-blood DNA methylation data and corresponding cellular composition measurements (e.g., flow cytometry) (Wiencke et al., 2017). This is not ideal because, in order to use IDOL, a new training data set needs to be created every time a reference signature is added for a new cell type. Further, IDOL also requires users to choose the size of the reference library in advance, which introduces arbitrary decisions in choosing a reference library.

Motivated by the shortcomings of these existing approaches, we propose an algorithm for reference library selection without a training dataset (RESET) that does not require users to choose the size of the reference library in advance, nor does it require a training data set like IDOL. Our approach is further driven by the desire to save researchers time and money; since a training data set does not need to be acquired or updated every time the reference signature for a new cell type becomes available. Further, choosing a reference library size in advance is an arbitrary decision that does not guarantee the quality of a library. RESET is similar in principle to IDOL but utilizes a modified version of the Dispersion Separability Criteria (DSC) as a metric for evaluating and ranking potential reference libraries for reference-based deconvolution. The Cancer Genome Atlas originally developed the DSC for detecting batch effects as a measure of the between vs. within batch dispersion (Akbani et al., 2020). We hypothesize that higher values of this metric correspond to better discrimination across cell types and, as a consequence, better prediction performance when used for reference-based deconvolution. In what follows, we first describe our Modified DSC metric and the proposed RESET framework for identifying reference libraries. We benchmark the performance of RESET compared to the Legacy method using three publicly available data sets across a range of different parameter settings. Next, we explore the impact that different libraries have on the operating characteristics of EWAS, using both simulations and data applications involving two publicly available data sets. We finish with a discussion of our findings, limitations of our study, and opportunities for future work.

## Materials and Methods

**FIGURE 1:**
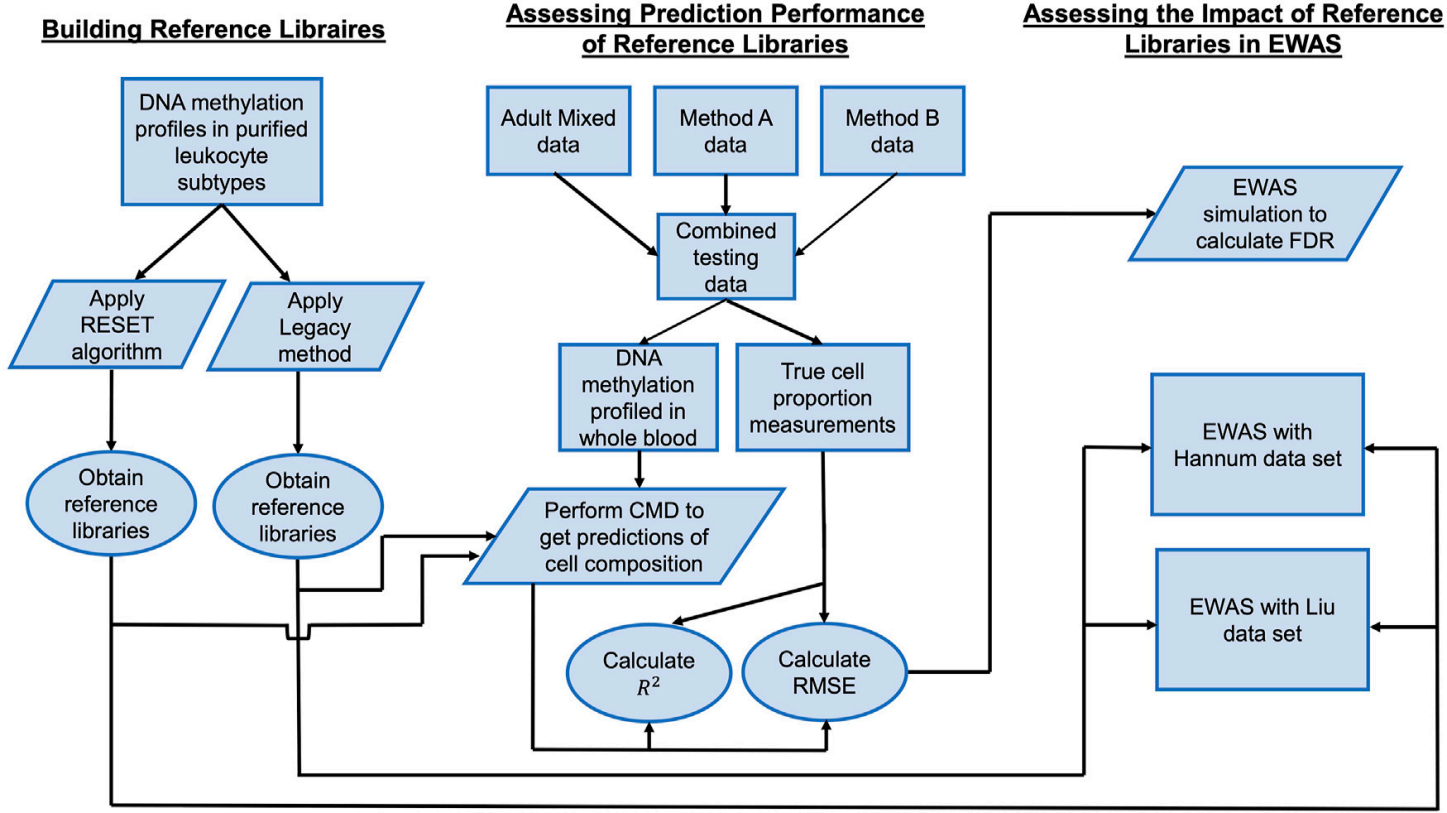
Analytical workflow. A workflow for describing the data sets used and analyses that were performed.

In what follows, we describe the materials and methods used in this research. Figure 1 provides a workflow that summarizes the data sets used and analyses that were performed.

### Cell Mixture Deconvolution

Before we introduce our proposed method, we first provide a brief description of cell mixture deconvolution. Let $Y_{i}=\left[Y_{i1},Y_{i2},\ldots ,Y_{iJ^{*}}\right]$ , where $0\leq Y_{ij}\leq 1$ , be the methylation beta-values for CpGs $j\in \left\{1,2,\ldots ,J^{*}\right\}$ for sample i∈{1,2,…,N} *.* Assume that for sample *i*, DNA methylation is profiled in a heterogeneous tissue type (e.g., whole-blood), which has *K* underlying cell types and whose proportions in sample *i* are denoted $w_{i}=\left[w_{i1},w_{i2},\ldots ,w_{iK}\right]$ . As previously described in Houseman et al. (2012), the methylation signature of $Y_{i}$ is assumed to be a weighted mixture of the DNA methylation signature of each of the *K* underlying cell types (Houseman et al., 2012). That is:

$$E\left[Y_{i}\right]=w_{i}\;\mu^{T},\;0\;\leq w_{ik}\leq 1\;\mathrm{and}\;\overset{K}{\underset{k=1}{\sum }}w_{ik}\;\leq 1$$

where μ is a $J^{*}$ x *K* matrix of mean methylation beta values. The rows of μ correspond to the same ordering of the $J^{*}$ CpGs as in $Y_{i}$ and the columns of μ are the *K* distinct cell types. Following the above equation, the Houseman approach to CMD proceeds by finding the $w_{i}$ that minimizes the squared-error loss function between $Y_{i}$ and $w_{i}\;\mu^{T}$ ,

$$argmin_{w_{i}}\|Y_{i}-w_{i}\;\mu^{T}{\|}^{2}$$

subject to the above-mentioned constraints on $w_{ik}$ . Since μ is unknown, the sample mean, **M**, is used and can be estimated using a reference methylation data set consisting of DNA methylation data on isolated, purified cell populations (Houseman et al., 2012; Reinius et al., 2012).

CMD is made possible by leveraging the unique methylation signature of each cell type, which is reflected in the columns of **M**. However, as previously described, the accuracy of cell proportion estimates, ${\hat{w}}_{i}$ , rely heavily on the $J^{*}$ CpGs are used as the basis of CMD. Strong reference libraries for CMD are comprised of CpGs whose methylation signature is distinct across each of the *K* cell types.

### Modified Dispersion Separability Criteria

To be able to perform CMD with high accuracy when the true cell proportions are not known, we need a metric that identifies $J^{*}$ CpGs that discriminate between cell types well. We believe that the Dispersion Separability Criteria (DSC) could be one such metric since it was designed by the Cancer Genome Atlas (TCGA) to identify batch effects in high-dimensional molecular data sets (Akbani et al., 2020). Defined as follows,

$$DSC=\;\frac{D_{b}}{D_{w}}$$

$D_{b}$ is a measure of dispersion between batches and $D_{w}$ is a measure of dispersion within batches. $D_{b}$ can be thought of as the mean Euclidean distance between batch centroids and the overall centroid, while $D_{w}$ can be thought of as the mean Euclidean distance between samples of a given batch and the centroid for that batch. More specifically $D_{b}$ and $D_{w}$ are defined as:

$$D_{b}=\;\sqrt{trace\left(S_{b}\right)}$$

$$D_{w}=\;\sqrt{trace\left(S_{w}\right)}$$

where $S_{b}$ is the between batch scatter matrix and $S_{w}$ is the within batch scatter matrix as described previously by Dy and Brodley (2004) (Dy, 2004). Higher DSC values indicate that there is a greater dispersion between batches than within batches and indicate the presence of batch effects. Thus, we hypothesized that for a given set of CpGs (e.g., library), high values of the DSC would indicate greater dispersion between cell types and that libraries with large DSC values correspond to improved deconvolution accuracy. However, our early exploration of this metric in selecting reference libraries for deconvolution found that this DSC and deconvolution accuracy is more complicated. Some cell types are more alike than others due to shared lineages. For example, the methylation pattern of CD8T and CD4T cells is more related than, say, CD8T cells and monocytes because of their shared lymphoid lineage. The above-mentioned formulation of the DSC was ill-suited to address such challenges, and poor deconvolution performance ensued.

To address such challenges, we propose a metric for assembling reference libraries for CMD that involves a slight modification of the DSC. We define the Modified DSC as follows:

$$Modified\;DSC=\;\frac{Min\left(D_{b}^{*}\right)}{Min\left(D_{w}^{*}\right)}$$

**FIGURE 2:**
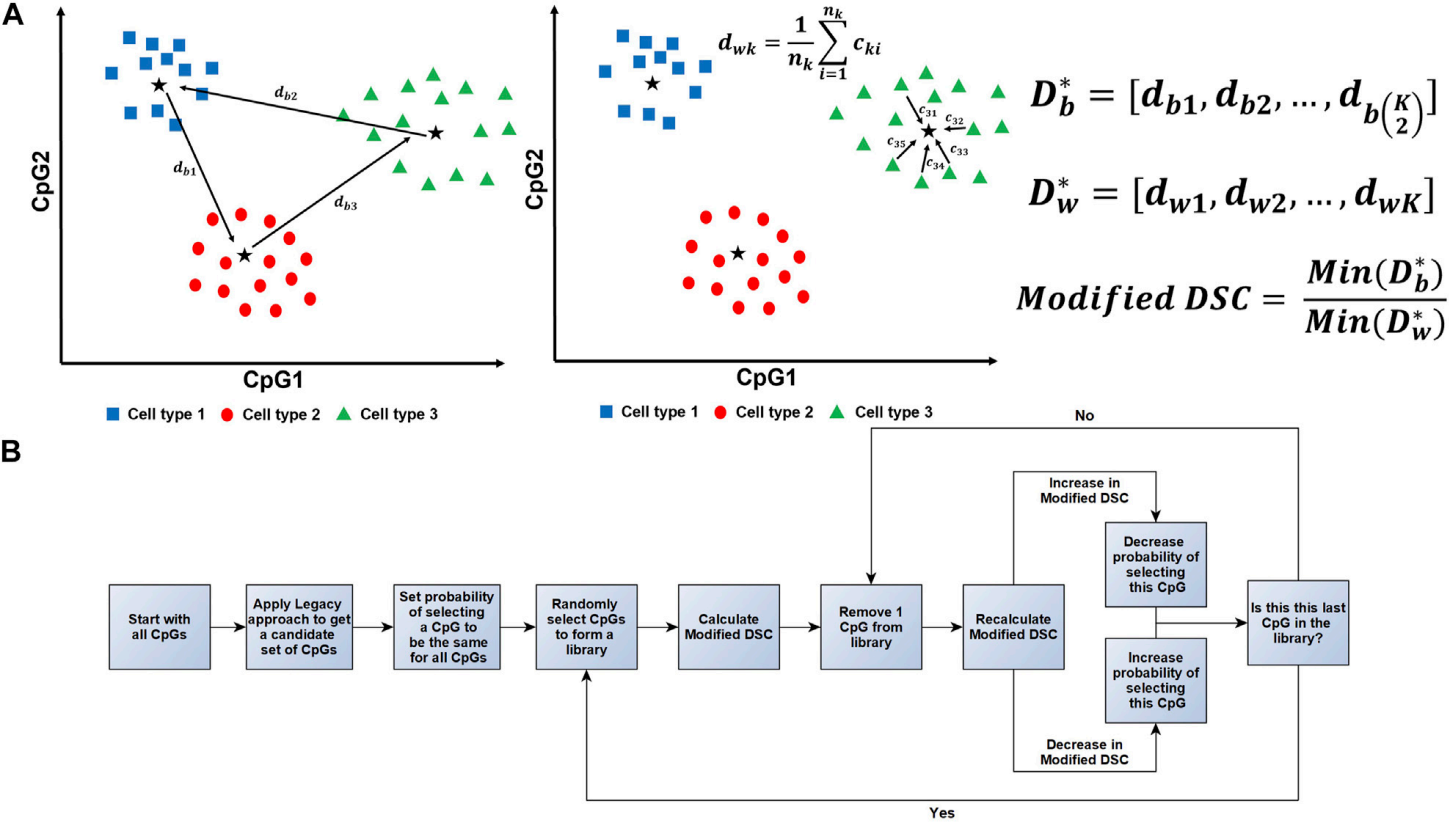
Illustration of the Modified DSC Calculation and Workflow. **(A)** A conceptual illustration of how the Modified DSC is calculated assuming three different cell types and two CpGs. (**A, Left**) $\;d_{b1},\;d_{b2}$ and $d_{b3}$ represent the Euclidean distances between pairs of cell-type-specific centroids (denoted as stars). These distances are elements of the vector, $D_{b}^{*}$ , which represents the between-cell type dispersion index. (**A, Right**) $c_{ik}$ , k=1,2,…,K and $i=1,2,\ldots ,n_{k}$ , represent the Euclidean distances between each sample of a given cell type and the centroid (denoted as star) for that cell type where *k* is an index for cell type, and *i* is an index for sample. The average within-cell type distance is represented by $d_{wk}$ , where $d_{wk}=\;\frac{1}{n_{k}}\overset{n_{k}}{\underset{i=1}{\sum }}c_{ik},\;\;$ and $n_{k}$ represents the number of samples for cell type *k*. These distances are the elements of the vector, $D_{w}^{*}$ , which represents the within-cell type dispersion index. The modified DSC is then calculated as the ratio of the minimum between-cell type dispersion and the minimum within-cell type dispersion, $\frac{Min\left(D_{b}^{*}\right)}{Min\left(D_{w}^{*}\right)}$ **(B)** Workflow for RESET algorithm to find the optimal library using the Modified DSC metric as described in *Algorithm for RESET Utilizing the Modified DSC Metric*.

$D_{b}^{*}$ represents a vector of the Euclidean distances between every pair of cell type centroids and $D_{w}^{*}$ is a vector containing the mean Euclidean distances between samples of a given cell type and the centroid for that cell type. More precisely:

$$D_{b}^{*}=d_{b1},d_{b2},\ldots ,d_{b[\begin{array}{c} K\\ 2 \end{array}]}$$

$$D_{w}^{*}=\left[d_{w1},d_{w2},\ldots ,d_{wK}\right]$$

where $\left(\begin{array}{c} K\\ 2 \end{array}\right)$ reflects the number of cell-type pairs. Although reference libraries for CMD are typically comprised of hundreds or thousands of CpGs, Figure 2A provides a simplified, conceptual illustration of the calculation of the Modified DSC, assuming three cell types and two CpGs. We chose to utilize the minimum of these distances after exploring various combinations of summary statistics (data not shown). The idea is that high values of the Modified DSC correspond to better discrimination between cell types since it is based on minimum distances. More specifically, if the minimum distance between any two cell type centroids is large and the minimum within-cluster distance is small, this will yield a large Modified DSC and indicate large differences between cell types with respect to their DNAm signature.

### Algorithm for RESET Utilizing the Modified DSC Metric

The below algorithm was taken from Koestler et al. (2016) and adjusted to utilize the Modified DSC when a training data set is not available (Koestler et al., 2016). A further illustration of this algorithm can be found in Figure 2B.

Step 0: Assembling a candidate reference library:
  a. Fit a series of two-sample t-tests to the *J* CpGs to compare mean methylation (beta-values) between each cell type against the mean beta-values across the remaining *K*-1 cell types.
  b. Identify $\frac{L}{2}$ CpGs with the largest t-statistics and the $\frac{L}{2}$ CpGs with the smallest t-statistics for each of the *K* cell types. Here *L* represents the number of cell-specific DMLs we desire.
  c. Create a set of CpGs, *Q*, identified in (b). Specifically, *Q* is comprised of *P* = *LK* DMLs and makes up the candidate search space for the subsequent steps.

It should be noted that the choice of *L* is completely arbitrary, and trade-offs must be made depending on how large *L* is chosen to be. Large values of *L* broaden the search space for possible cell-specific DMLs, but also increase the computational burden in downstream steps. Small values of *L* correspond to less computational burden but risk excluding viable cell-specific DMLs.

Step 1: Random assembly of reference libraries:
  a. At iteration ℓ *,* $J^{*}$ CpGs are randomly chosen from *Q* with probability $\pi_{j}^{\ell },\;j\in \left\{1,\ldots ,P\right\}$ . At the first iteration, ℓ=0 , every CpG among the *P* candidate DMLs has an equal probability of being chosen. That is, $\forall \;j\;\in \left\{1,\ldots ,P\right\},\;\pi_{j}^{0}=\;\frac{1}{P}\;.$

  b. Let $Q^{\ell }\subset Q$ denote the randomly assembled reference library, comprised of $J^{*}$ randomly selected CpGs at iteration ℓ .

Note that $J^{*}$ , the number of CpGs that make up the library $Q^{\ell }$ , is also randomly selected from a uniform distribution with a minimum of $j_{min}\geq k$ and a maximum of $j_{max}\;\leq P$ .

Step 2: Calculation of the Modified DSC using the randomly assembled library:
  a. Using the random library $Q^{\ell }$ , calculate the Modified DSC. We will denote this $MDSC^{\ell }.$

Step 3: Assessing the contribution of each CpG to the Modified DSC using leave-one-out procedure:
  a. Each of the $J^{*}$ CpGs that make up $Q^{\ell }$ are iteratively removed to obtain sets of CpGs, $Q_{-j}^{\ell },$ which includes all CpGs in $Q^{\ell }$ , except CpG *j*.
  b. Step 2 is repeated for each reduced library $Q_{-j}^{l}$ so that we have $J^{*}$ new Modified DSCs, denoted as $MDSC_{-1}^{\ell },\;MDSC_{-2}^{\ell },\;\ldots ,\;MDSC_{-J^{*}}^{\ell }$ .

In subsequent iterations, we seek to retain CpGs whose inclusion in the reference library results in large values of the Modified DSC. Calculating $MDSC_{-j}^{\ell }$ gives us a framework for updating selection probabilities of each CpG in. $Q^{\ell }.$

Step 4: Updating selection probabilities:
a. For each of the $J^{*}$ CpGs in the reduced library, $Q^{\ell }$ , we update the probability of selecting CpG *j* as follows:

$$r=\;\frac{MDSC^{\ell }}{MDSC_{-j}^{\ell }}$$

$$\pi_{j}^{\ell +1}=\;\pi_{j}^{\ell }\;r$$

Thus, the greater the contribution CpG *j* has on the value of the Modified DSC, the more influence it has on the probability of being selected in future iterations. More specifically, if removing CpG *j* from $Q^{\ell }$ results in a smaller value of the Modified DSC, *r* will be greater than 1 and thus $\pi_{j}^{\ell +1}$ will increase. If removing CpG *j* from $Q^{\ell }$ results in a larger value of the modified DSC, *r* will be less than 1 and thus $\pi_{j}^{\ell +1}$ will decrease. How much $\pi_{j}^{\ell }$ changes from iteration to iteration depends on the contribution of CpG *j* to the Modified DSC.

b. After the probabilities for the $J^{*}$ CpGs in the random library, $Q^{\ell }$ , have been updated, the probabilities of all *P* CpGs in *Q* are then scaled to sum to 1.
Step 5: Continue iteration*:* Using the updated probabilities, $\pi_{j}^{\ell +1}\;j\in \left\{1,\ldots ,P\right\}$ , repeat steps 1–4. The final solution is the library made up of the $J^{*}$ CpGs, which yielded the largest value of the Modified DSC.

### Datasets

We now describe the DNA methylation array data sets used for this research. For more information about how the data was preprocessed and what measures were taken to ensure quality control, we refer readers to Koestler et al. (2016).

#### Cell Mixture Reconstruction Experiment

Purified cells taken from normal human subjects were purchased from AllCells LLC (Emeryville, CA). Namely, granulocytes, monocytes, CD4T, CD8T, natural killer, and B cells. DNA was extracted from the purified blood leukocyte subtypes, dsDNA was quantified, and then DNA extracted from purified leukocyte subtypes were mixed in preplanned proportions to reconstruct two different sets with *n* = 6 samples in each. One set of samples was reconstructed to contain similar proportions of the purified leukocyte subtypes. In contrast, the other was reconstructed to resemble the proportions in normal human adults’ peripheral blood. We will refer to these data sets as MethodA and MethodB, respectively. These DNA samples were bisulfite modified, and epigenome-wide DNA methylation assessment was done using the Illumina HumanMethylation450 array platform (Koestler et al., 2016). These data are publicly available in the Gene Expression Omnibus (GEO) repository (GEO Accession ID: GSE77797).

#### Adult Whole Blood Samples

Another *n* = 6 whole blood (WB) samples were taken from healthy adult donors. Immune cell profiling data for these samples, gathered from flow cytometry, were procured from AlCells LLC. We will refer to this data set as AdultMixed. This data set followed the same protocol as above and was assayed using the Illumina HumanMethylation450 array platform (Koestler et al., 2016). These data are publicly available (GEO Accession ID: GSE77797).

#### Reference DNA Methylomes for Isolated Leukocyte Subtypes

A publicly available dataset (GEO Accession ID: GSE35069) was used to identify cell-type-specific DMLs and for the construction of reference libraries for CMD. This data set is comprised of epigenome-wide DNA methylation profiles in purified leukocyte subtypes (the same six leukocytes described above) in *n* = 6 healthy non-diseased subjects. We will refer to this data set as the Reference data set. Further details about this data set can be found elsewhere (Reinius et al., 2012).

#### Additional DNA Methylation Data Sets

We also used two large publicly available blood-derived DNA methylation data sets (GEO Accession IDs: GSE42861 and GSE40279). Together, these two data sets are comprised of whole-blood DNA methylation data on more than 1,200 adult subjects. The purpose of these data sets was to better understand the implications of the method/technique used for assembling reference libraries for CMD in terms of an EWAS. The Liu dataset (GEO Accession ID: GSE42861) consists of blood-derived DNA methylation data on 689 human subjects, of which *n* = 354 were rheumatoid arthritis cases, and *n* = 335 were non-diseased control patients (Liu et al., 2013). The Hannum data set (GEO Accession ID: GSE40279) consists of blood-derived DNA methylation on 656 non-diseased patients (Hannum et al., 2013). Epigenome-wide DNA methylation assessment was done using the Illumina HumanMethylation450 array platform for both data sets.

### Application and Assessment of the Modified DSC Metric

#### Proof-Of-Principle Example to Illustrate the Modified DSC Metric

To examine our hypothesis that larger values of the Modified DSC reflect libraries that better discriminate cell types, we conducted a proof-of-principle simulation study where we simulated data under different circumstances and calculated the Modified DSC. We simulated 100 features for three different cell types with six samples of each cell type. We allowed the percentage of features that exhibit a difference across the cell types to vary between 0% and 100%. We further allowed for the magnitude of the difference in the mean feature values to vary between 1 and 10 for each cell type. Let $Y_{jk},$ be the value for feature j, j∈{1,2,…,100} for cell type k, k∈{1,2,3} . For the features that were randomly selected to exhibit a difference across cell types, we randomly chose one cell type to have mean 0, one cell type to have mean Δ , and one cell type to have mean −Δ , where Δ reflects the magnitude of the difference between the mean feature levels. Thus, these features were distributed as $Y_{i1}∼Normal\left(0,1\right),\;Y_{i2}∼Normal\left(\Delta \;,1\right)$ , $Y_{i3}∼Normal\left(-\Delta \;,1\right)$ . All other features were distributed as $Y_{ik}∼Normal\left(0,1\right)$ across the three cell types. While DNA methylation data is not normally distributed, the design implemented here nevertheless emulates the behavior of cell-specific methylation and provides a useful framework for understanding the behavior of the Modified DSC as a function of the number/fraction of distinct features and the magnitude of their difference across cell types. After each dataset was simulated, the Modified DSC was calculated. Finally, a heatmap of simulated data and a plot of the first two principal components (PC1 vs. PC2) were generated to visualize the separation of the cell types for a library with no differences, a library with moderate cell-specific differences, and a library with large cell-specific differences.

#### Training the Algorithm

To examine the performance of RESET, we conducted an analysis of several publicly available data sets and benchmarked the accuracy of deconvolution estimates obtained using RESET against the Legacy approach. To do this, we first applied our approach using the Reference data set to identify “optimal” reference libraries for deconvoluting WB mixtures. As previously described, the reference data set is comprised of DNA methylation profiles for granulocytes, monocytes, CD4T, CD8T, natural killer, and B cells in *n* = 6 subjects. To get a candidate list of CpGs, we first applied the Legacy approach to the broader reference data set. We specified *L* to be 50, giving us the top 50 hyper and hypomethylated CpGs for each cell type for a total of 1800 CpGs ( J=1800 ). After this, we ran RESET for 100,000 iterations. The library that yielded the largest value of the Modified DSC was taken to be the “optimal” reference library and used for subsequent CMD. A workflow for this analysis can be found in Figure 2B. We also applied the Legacy approach, as previously described, to the same reference data set. To compare the prediction performance across a range of library sizes, we assumed different values for $J^{*}$ . That is, we fixed $J^{*}=72,\;120,\;180,\;240,\;300,\;360,\;540$ for both RESET and the Legacy method. Further, since the Legacy approach and IDOL require users to specify the size of the reference library, we also analyzed prediction performance for RESET when $J^{*}$ was treated as random. Specifically, instead of fixing $J^{*}$ to be one of the values above, we let $J^{*}$ be a random number between 50 ( $j_{min}$ ) and 1,500 ( $j_{max}$ ). As before, the library that resulted in the largest value of Modified DSC was taken to be the “optimal” reference library. It should be noted that, currently, the only other method for building reference libraries, which does not require a training data set, is the ANOVA approach. As this method has been shown to perform poorly compared to the Legacy approach in Koestler et al. (2016), we only benchmark RESET against the Legacy approach (Koestler et al., 2016).

#### Validation of the Identified Reference Libraries

To validate RESET, we applied CMD to three independent testing sets (MethodA, MethodB, AdultMixed) using the libraries identified using the RESET algorithm. These data sets were selected since the underlying leukocyte fractions are known for each sample. Figure 4A illustrates the differing cell-type proportions across the three testing data sets.

To assess the performance of our method and the Legacy approach, we first estimated the proportion of variation of the known mixture fractions explained by the cell type predictions ( $R^{2}$ ). We also assessed our performance by calculating the root mean squared error (RMSE), which reflects the average deviation between the true mixture proportions and our predictions. These metrics were computed for each cell type individually as well as their averages across all cell types for each reference library we obtained.

#### Simulation Study Comparing the FDR

To explore the implications of prediction error in cell proportion estimates for EWAS, we conducted a simulation study in which the false discovery rate (FDR) was calculated for cell composition adjusted EWAS, where cell composition estimates were based on differing methods/approaches for library selection. We implemented a study design that is common to many EWAS, in which the goal is to identify differently methylated CpGs between two groups of samples ( $n=\;n_{1}+n_{2}$ ). We conducted this simulation considering a moderately sized study ( $n_{1},n_{2}=100$ ) and assuming a large study ( $n_{1},n_{2}=500$ ). Further, we introduced varying dissimilarities in the true, simulated cellular distribution between the cases and controls.

For this simulation study, we assumed that the methylation beta-value for CpG *j* for sample *i*, that is $Y_{ij},$ follows a beta-distribution with mean $w_{i}\mu_{j}^{T}$ and variance $\frac{\left(1-w_{i}\mu_{j}^{T}\right)w_{i}\mu_{j}^{T}}{1+\;\phi_{j}}$ Recall that $w_{i}$ is a vector that represents the true cell proportions for sample *i*, $\mu_{j}$ is a vector of mean methylation beta-values for CpG *j* across the *K* cell types, and $\phi_{j}$ is the unobserved precision parameter for CpG *j* where $\phi_{j}>0$ . Often, EWAS fit regression models as follows:

$$Y_{ij}=\;\alpha_{0j}+\alpha_{1j}X_{i}+\overset{K-1}{\underset{k=1}{\sum }}\gamma_{kj}w_{ik}+\epsilon_{ij},$$

$$E\left[\epsilon_{ij}\right]=0\;and\;Var\left[\epsilon_{ij}\right]=\;\sigma_{j}^{2}$$

Here, $\overset{K-1}{\underset{k=1}{\sum }}\gamma_{kj}w_{ik}$ controls for cell composition across subjects, $X_{i}\in \left(0,1\right)$ denotes the group subject *i* belongs to, and $\epsilon_{ij}$ captures any remaining variation in methylation after controlling for group and cell proportions. Usually, interest lies in tests of no difference in DNA methylation between groups. That is, testing the null hypothesis that $\alpha_{1j}=0$ . In practice $w_{ik}$ is unknown and is estimated by ${\hat{w}}_{ik},$ which is obtained through CMD (Houseman et al., 2012). However, ${\hat{w}}_{ik}$ is an estimate, so any hypothesis tests based on the above regression model can be susceptible to Type 1 and Type 2 errors (Koestler et al., 2016).

To check how cell proportion prediction errors estimated using the RESET and Legacy libraries affect the FDR of hypothesis tests ( $H_{0}:\;\alpha_{1j}=0$ ), we first estimated the uncertainty of cell proportion predictions for each method. To do this, we squared the RMSEs across the three testing data sets, across the different library sizes, to get mean squared prediction errors (MSPEs). We did this for both the Legacy approach and RESET. We will denote the MSPEs as ${\hat{\tau }}_{kl}^{2}$ , where *l* represents the type of library used (either Legacy or RESET), and *k* is the index for cell type. To estimate the mean and precision parameters, we fit Beta regression models to each CpG. We then conducted our simulation study by randomly sampling 1,000 CpGs, generating random cell distributions for the two groups, simulating beta-values for each group, randomly sampling cell type predictions for each sample, fitting the above regression model to each of the CpGs, and calculating the FDR for each method assuming a nominal *p*-value cut off of 0.05. More specific details about the algorithm for this simulation can be found in Supplementary Material S1. Recall that the methylation beta-values for the two groups were simulated assuming no difference in the methylation profile (e.g., under the null hypothesis $\;\alpha_{1g}=0$ ). Thus, the only difference between the two groups lies in the dissimilarity in cell composition. So, any rejections of the hypothesis of $\;\alpha_{1g}=0$ reflect a Type 1 error.

#### Data Application and Implications for EWAS

We finally used the Liu and Hannum data sets described above to understand the implications of the Legacy approach and the RESET algorithm on cell type prediction for EWAS (Hannum et al., 2013; Liu et al., 2013). Specifically, an analysis of these blood-derived DNA methylation data sets was done to see which method performed better in explaining variation in DNA methylation. To do this, we started by using CMD for estimating cell proportions in the two data sets using the reference libraries identified using the RESET method, as well as the libraries identified using the Legacy approach. For each data set, linear regression models were fit to the *J* total CpGs independently, modeling beta-values as the response against the predicted cell proportions. Using the fitted models, we found the proportion of variation in methylation explained by the cell mixture proportion estimates using both methodologies ( $R_{j}^{2},\;j\in \left\{1,\;2,\;\ldots ,\;J\right\})$ . The difference in $R^{2}$ between models, adjusted for cell composition estimates using RESET and the Legacy approach were then calculated for each of the *J* CpGs. That is, $\Delta_{j}=\;R_{j,RESET}^{2}-R_{j,\;Legacy}^{2}$ Finally, to answer our question, we computed the proportion of CpGs where RESET explained more variation in the methylation explained as compared to the Legacy approach ( $\frac{1}{J}\overset{J}{\underset{j=1}{\sum }}I\left(\Delta_{j}\;>\;0\right)$ ) for each library size.

## Results

### Proof-Of-Principle Example to Illustrate the Modified DSC Metric

**FIGURE 3:**
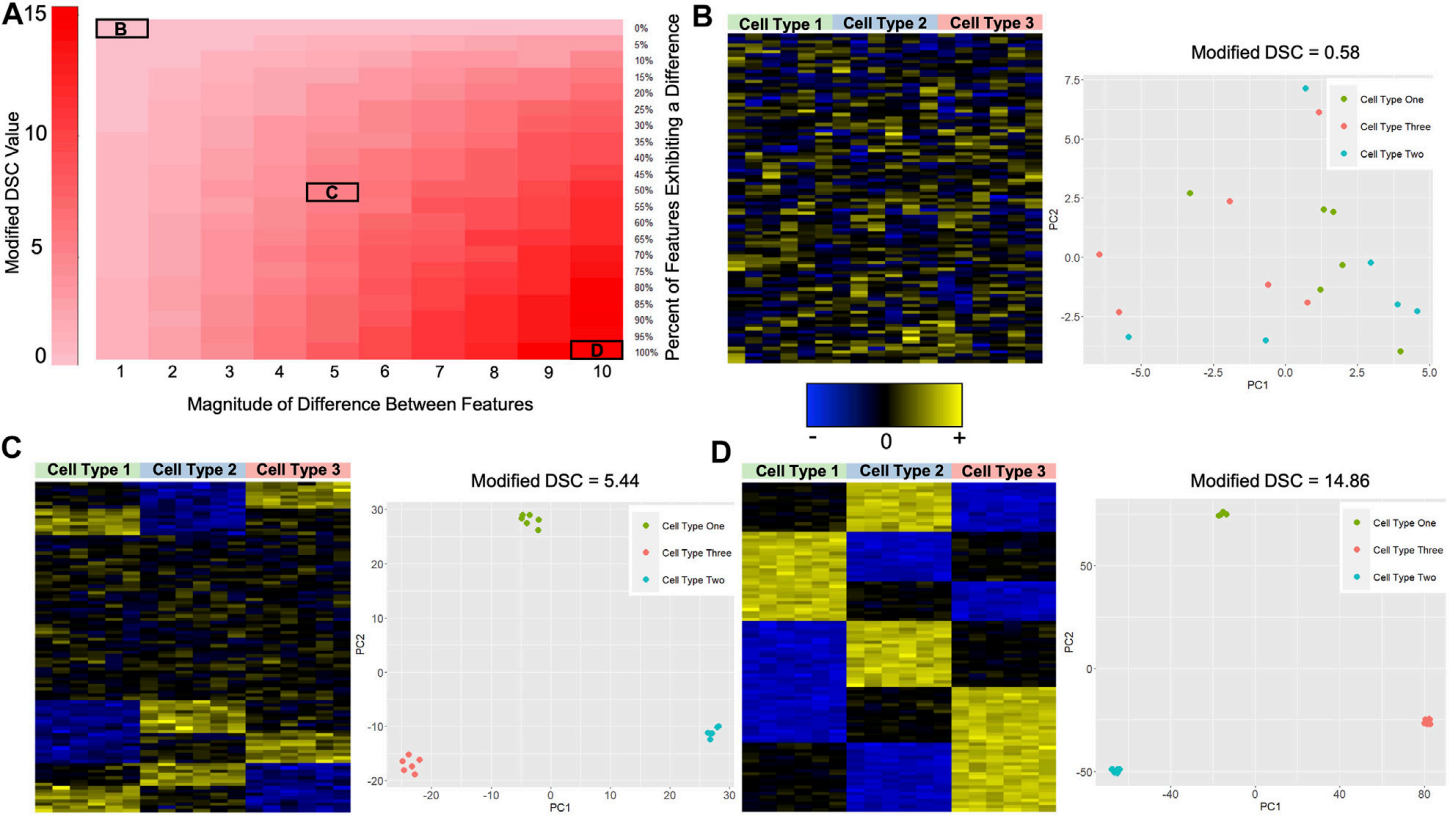
Proof-of-Principle Illustration of the Modified DSC Metric. **(A)** Heat map showing the value of the Modified DSC under the different data scenarios as described in section 2.5.1. The *y*-axis shows the percent of features that were simulated to exhibit a difference in the mean feature(s) value across cell types, and the *x*-axis reflects the magnitude of the difference between the mean feature(s) values across cell types. Darker red indicates higher values of the Modified DSC. **(B)** Heat map and PCA plot for the simulated data where no features exhibited a difference in the mean feature(s) values across cell types. The value of the modified DSC for this simulated data is also provided. **(C)** Heat map and PCA plot for the simulated data where 50% of the features exhibited a difference in mean feature(s) values across cell types with a magnitude of 5 (i.e., Δ=5 ). The value of the modified DSC for this simulated data is also provided. **(D)** Heat map and PCA plot for the simulated data where 100% of the features exhibited a difference in the mean feature(s) values across cell types with a magnitude of 10 (i.e., Δ=10 ). The value of the modified DSC for this simulated data is also provided.

To rationalize the use of the Modified DSC as a metric for identifying sets of CpGs which discriminate well between cell types, we simulated cell-specific methylation data with varying degrees of differences and calculated the Modified DSC. Recall that we allowed the percentage of features that exhibit a difference across the cell types to vary as well as allowing the magnitude of the difference in mean methylation to vary across cell types. Each of these differences gave us a new cell-specific methylation data set. We hypothesized that higher values of the Modified DSC result in reference libraries that better discriminate the cell types. Figure 3A shows the results for the 210 different simulated data sets. As the number of features/CpGs that exhibit a difference between cell types increases, and the magnitude of this difference increases, the value of the Modified DSC gets larger. The minimum Modified DSC value of 0.580 occurred when 0% of the 100 features were simulated to exhibit a difference across the three cell types. The maximum DSC value of 15.252 occurred when 90% of the 100 features were simulated to exhibit a difference across the cell types with a magnitude of 10, the largest possible magnitude difference considered in our simulation study. To further demonstrate the utility of the Modified DSC for building reference libraries for cell mixture deconvolution, we looked at heat maps of the simulated data and plots of the first two principal components (PCs) calculated from a principal components analysis (PCA) of the simulated data at various degrees of differences. Figure 3B depicts the data that were simulated to exhibit no difference in feature/CpG values across the three cell types. Not surprisingly, the heat map shows no discernible difference between cell signatures. Further, no discernable clustering samples by cell type are evident in the plot of PC1 by PC2. The value of the Modified DSC for this data set is 0.580. Figure 3C depicts the data set where 50% of the features/CpGs were simulated to exhibit a magnitude difference of 5 between the three cell types. The heat map shows some differences in the signatures across cell types, and the plot of PC1 by PC2 shows a clear separation of samples by cell type. The value of the Modified DSC for this data is 5.443. Finally, Figure 3D depicts the data that were simulated to exhibit a difference in 100% of the features/CpGs, with that difference being 10. The heatmap shows clear cell-type-specific signatures, and the plot of PC1 by PC2 shows even more separation in the clusters of cell types (take note of the scale of the *x*- and *y*-axis in the PC-plots between Figures 3C,D).

### Validation of the Optimal Reference Libraries

**FIGURE 4:**
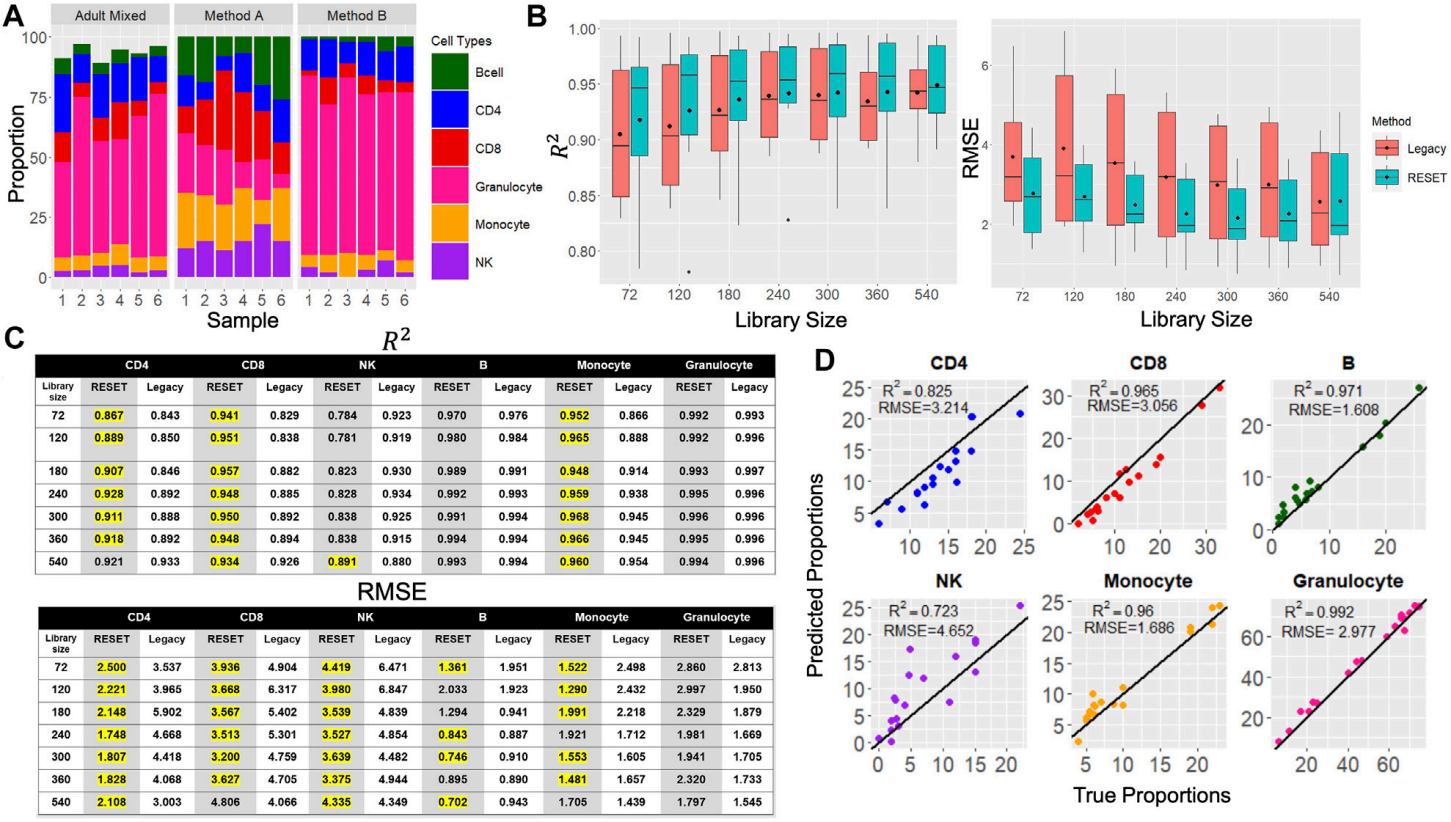
Results from RESET and Legacy Approach. **(A)** True cell-type proportions for the three independent data sets used to generate predictions for the RESET and Legacy approaches. Each data set is comprised of six samples and the same six leucocyte subtypes. **(B)** Box plots showing the $R^{2}$ and RMSE across all cell types for each assumed reference library size (recall $J^{*}=\left\{72,\;120,\;180,\;240,\;300,\;360,\;540\right\}$ ) for RESET compared to the Legacy approach. **(C)** Table of results comparing the prediction performance of the RESET algorithm and the Legacy approach for each cell type and across different assumed reference library sizes (rows of the table). The top table shows the $R^{2}$ while the bottom table shows the RMSE, calculated by comparing the predicted/estimated versus observed cell proportions. Yellow highlighted text denotes the conditions where the use of RESET resulted in better prediction performance than the Legacy approach. **(D)** Cell-specific scatter plots of the observed (*x*-axes) versus estimated (*y*-axes) cell proportions using the reference library yielded the largest value of the Modified DSC when the number of DMLs that comprise the library was allowed to be random. Additionally, the $R^{2}$ and RMSE for these predictions are provided.

As mentioned previously, to validate the reference libraries identified by RESET, we examined CMD prediction performance by calculating $R^{2}$ and RMSE using three independent test sets: Method A, Method B, and Adult Mixed, and benchmarked the performance of RESET against the Legacy approach for building reference libraries. As noted in Figure 4A, the different testing data sets are made up of varying proportions of each cell type. The six samples in the Method A testing set have approximately equal proportions of each cell type with the mean proportion as follows: CD4T = 11.8%, CD8T = 20.8%, NK = 15%, B = 16%, Monocyte = 19.2%, Granulocyte = 17.2% (Wiencke et al., 2017). The Method B samples more closely resemble the immune cell landscape in human adults with mean proportions as follows: CD4T = 13.2%, CD8T = 6%, NK = 3%, B = 2.7%, Monocyte = 6.2%, Granulocyte = 69% (Wiencke et al., 2017). Like the Method B samples, the Adult mixed samples had mean proportions as follows: CD4T = 17.9%, CD8T = 9.7%, NK = 3.5%, B = 4.9%, Monocyte = 6.7%, Granulocyte = 57.4% (Wiencke et al., 2017).

Prediction performance for RESET and the Legacy method was evaluated for several different library sizes ( $J^{*}=72,\;120,\;180,\;240,\;300,\;360,\;540$ ) using the three mentioned testing data sets. RESET resulted in categorically better prediction performance over the Legacy approach. The left plot of Figure 4B shows the $R^{2}$ across the six cell types for each library size. On average RESET only did marginally better than the Legacy method. However, the median $R^{2}$ for RESET was higher than Legacy for all library sizes, except for 540. Improvements in prediction performance were even more apparent when looking at the RMSE. The right plot of Figure 4B shows the RMSE across the six cell types for each library size. On average, the RMSE was smaller when using our approach for all library sizes except for 540. Further, median RMSE was smaller for all library sizes when using RESET.

Cell-type-specific $R^{2}$ and RMSE values for both RESET and the Legacy method can be found in Figure 4C. Interestingly, for CD4T, CD8T, and monocytes the $R^{2}$ was higher than what was observed in the Legacy approach across nearly all reference library sizes considered. The $R^{2}$ for granulocytes and B cells was very close to 1 for both our method and the Legacy approach. However, the $R^{2}$ was higher for the Legacy approach in NK cells. Additionally, the RMSE was lower for nearly all reference library sizes using RESET for CD4T, CD8T, NK, B, and monocytes. The only cell type that did not have lower RMSE when comparing the libraries constructed using the Modified DSC approach to the Legacy method was granulocytes.

In addition to comparing the $R^{2}$ and RMSE for the fixed libraries, we also calculated the Modified DSC value across the RESET land Legacy libraries. The average difference in the Modified DSC across the fixed libraries for the two methods was 14.82, indicating that on average, the Modified DSC of libraries obtained via RESET is higher than the Legacy method.

When the reference library size was chosen randomly, RESET identified a library with 54 CpGs. That is, the library size that resulted in the largest value of the Modified DSC was comprised of 54 loci. The prediction performance was evaluated using the same three previous testing data sets. Even with so few CpGs, this library still had an average $R^{2}$ of 0.906 across all six cell types and an average RMSE of 2.865. CD8T, B, monocyte, and granulocyte predictions all had an $R^{2}$ higher than 0.950. The predictions for NK and CD4T cells had an $R^{2}$ of 0.723 and 0.825, respectively. Figure 4D depicts the predicted cell proportions versus the true cell proportions for each of the six cell types based on this reference library.

### Simulation Study Comparing FDR

**FIGURE 5:**
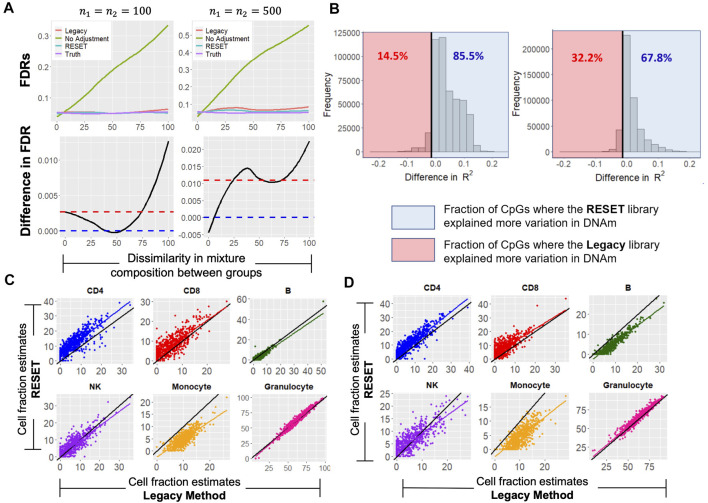
FDR Simulation Results and Data Application Results for the DML Reference Library of Size 120. **(A)** Results from the FDR simulation to explore implications of prediction error in cell proportion estimates for EWAS as described in section 2.5.4. The top panel shows the estimates of the FDR for the two-group comparison of methylation as a function of the dissimilarity in the distribution of cell-type composition between the two groups. The FDRs were estimated under four methods/approaches for cell composition adjustment. The bottom panel shows the difference in FDR estimates when cell type estimation was undertaken using the Legacy library versus the RESET library. Points that lie above the blue dotted line signify that the Legacy library resulted in more false-positive results as compared to the RESET. The red dotted line shows the mean difference in FDR between the Legacy library and the RESET library. A loess smoother was used to generate these curves. **(B)** Distribution of the difference in $R^{2}$ obtained from the RESET and Legacy libraries of size 120 CpGs applied to the Liu (left panel) and Hannum (right panel) data sets described in section 2.5.5. **(C)** Scatter plots of the predicted cell-type proportions using the Legacy library of 120 CpGs (*x*-axis) and the RESET library of 120 CpGs (*y*-axis) as applied to the Liu data set. **(D)** Scatter plots of the predicted cell-type proportions using the Legacy library of 120 CpGs (*x*-axis) and the RESET library of 120 CpGs (*y*-axis) as applied to the Hannum data set.

The validation results showed that the libraries identified by RESET were overall only marginally better than those identified by the Legacy approach; however, prediction performance is often not an end in itself but rather a means to an end. Specifically, the cell proportion estimates identified from these libraries are often used for cell-type adjustment in downstream statistical analysis. As such, we performed a simulation to explore the consequences of prediction error in cell proportion estimates for EWAS (Figure 5A, top two plots). We conducted a simulation study where methylation beta values were simulated for two groups, 1,000 CpGs, and assuming both moderate and large within-group sample size (*n* = 100, 500, respectively). Additionally, the cellular composition varied across groups while the cell types were simulated to have no difference between groups. When adjustments for cell composition were made using the true simulated cell proportions, the FDR was controlled at 5% as expected. When no adjustments for cell composition were made, there was large inflation in the FDR, which increased as a function of increasing cell composition dissimilarity between groups. When n1 = n2 = 100, the FDR was controlled at 5% when adjustments for cell composition were made using both RESET and the Legacy approach. However, when n1 = n2 = 500, there was slight inflation in the FDR using the Legacy approach while it remained controlled at 5% when using RESET. Additionally, the bottom two plots of Figure 5A show the difference in FDR from the Legacy approach and our approach. RESET, on average, resulted in fewer false positives compared to the Legacy approach across the different simulation conditions.

### Data Application and Implications for EWAS

In addition to using the above simulation study, we also made use of two publicly available data sets to better understand the consequences of the approach used for reference library construction in the context of EWAS. All fixed reference libraries that were previously identified were applied to the Lui and Hannum data sets (Hannum et al., 2013; Liu et al., 2013) to see which libraries led to a better explanation in the variation in DNA methylation ( $R^{2}$ ). When using the Liu data set, the proportion of CpGs where RESET explained more variation in the methylation, as compared to the Legacy approach, was high for most of the library sizes. These proportions are 0.654, 0.855, 0.892, 0.871, 0.870, 0.882, and 0.774 for the library sizes of 72, 120.180.240, 300, 360, and 540, respectively. When using the Hannum data set, the proportion of CpGs where RESET explained more variation in the methylation, as compared to the Legacy approach, while not as high, was still greater than 0.5 for all libraries. These proportions are 0.537, 0.678, 0.728, 0.678, 0.680, 0.677, and 0.590 for the library sizes of 72, 120.180.240, 300, 360, and 540, respectively. A visualization of the results when using the reference libraries of size 120 is shown in Figure 5B. Scatter plots of the cell proportion estimates of the RESET library of size 120 compared to the estimates of the Legacy library of size 120 can also be found in Figure 5C (using the Liu data set) and Figure 5D (Using the Hannum data set). RESET tended to result in higher predicted proportions for CD4T and CD8T cells for both data sets while tending toward lower predicted proportions for B cells and monocytes. Similar visualizations for results using the other libraries can be found in Supplementary Material S2.

## Discussion

This manuscript described and evaluated an iterative algorithm for assembling libraries for reference-based deconvolution in the absence of a training dataset. The motivation for this research is three-fold. First, the performance of reference-based deconvolution depends heavily on the reference library used as the basis of deconvolution (Koestler et al., 2016; Teschendorff et al., 2017; Titus et al., 2017). Second, in the absence of a training data set, current approaches require users to make arbitrary decisions about the number of DMLs and the library size even though performance is sensitive to these decisions (Houseman et al., 2012; Koestler et al., 2016). Third, emergence and interest in new cell types means that not only will reference libraries need to be updated but training data sets also. If DNAm along with measured cell-type proportions for these new cell types are not available, then IDOL is not possible (Koestler et al., 2016).

Recall that RESET builds DNAm reference libraries by utilizing the Modified DSC. This metric increases in value as clusters of cell-type-specific DNAm data get farther apart from one another and makes it a useful metric for evaluating the quality of a reference library. RESET works by randomly selecting a reference library from a candidate list of CpGs, calculating the Modified DSC, and then iteratively updating the probability of selecting a CpG in future iterations depending on its contribution to the modified DSC. The library which yields the largest value of the Modified DSC after many iterations is the reference library used for deconvolution. As previously described, while the Legacy approach has been previously shown to outperform the ANOVA-based method for library construction (Koestler et al., 2016), its primary shortcoming is that the user needs to prespecify both the total library size and the number of cell-specific DMLs to include in the library. As shown here (Figure 3) and previously (Koestler et al., 2016), the accuracy of corresponding deconvolution estimates is sensitive to these selections, with the $R^{2}$ of some cell types varying by nearly 0.10 units across the limited range of library sizes considered here. This observation is not surprising as leukocytes share common linages, and thus varying numbers of DMLs may be needed for different leukocyte subtypes (Koestler et al., 2016). While the IDOL algorithm is an improvement on both the ANOVA-based and Legacy approaches, it requires a training data set consisting of both DNAm data—profiled in whole blood or some other heterogeneous biospecimen—and gold standard measurements of cellular composition in the same biospecimen used for methylation profiling (Koestler et al., 2016). Although such data presently exist for whole blood (Reinius et al., 2012; Koestler et al., 2016), they typically only include relative fractions of the major leukocyte components of whole blood (e.g., CD4T, CD8T, B cells, NK cells, monocytes, and granulocytes) and lack measurements of more exotic cell types (e.g., monocytic and/or granulocytic myeloid-derived suppressor cells) and cells with specific activation states (e.g., CD8T naïve, central memory, and effector memory). In contrast to the ANOVA-based, Legacy, and IDOL approaches, RESET does not require a training data set, the number of DMLs for each cell type need not be the same, and the total number of loci that make up the reference library does not need to be specifically selected in advance.

While our results showed a modest improvement in the average $R^{2}$ and RMSE resulting from the libraries constructed using RESET compared to the Legacy approach, they showed considerable improvement in the accuracy for specific cell types (CD4T, CD8T, and monocytes). For these three cell types, the libraries identified using RESET had a larger $R^{2}$ than those identified by the Legacy approach in all but one library (Figure 4C). Additionally, the RMSE for CD4T, CD8T, and monocytes were smaller using RESET libraries in all but two cases (Figure 4C). Our algorithm seemed to favor choosing small libraries for the library identified using a random number of loci. That is, the random-sized reference library with the largest value of the Modified DSC was comprised of only 54 CpGs. The $R^{2}$ averaged across all six cell types for this library was 0.906. This was smaller than the average $R^{2}$ for most of the Legacy libraries, but within 0.04 units. Interestingly, the CD8T and monocytes cell-specific $R^{2}$ were higher than any of the cell-specific $R^{2}$ for the Legacy libraries. Further, the average RMSE for this random library was 2.865, which was better than all but one of the Legacy libraries. Also interesting is the performance of this library when compared to the performance of the library identified by IDOL. The IDOL library was comprised of 300 loci, had an average $R^{2}$ of 0.993 across the six cell types, and an average RMSE of 1.15 (Koestler et al., 2016). While, on average, IDOL outperforms our algorithm, we note that IDOL should be expected to be slightly better because it utilizes a training data set. The slightly lower prediction performance of RESET is made up by the fact that our algorithm does not need a training data set. In addition to the favorable performance of the randomly sized library, there are also downstream benefits to having a small reference library for deconvolution. First is the fact that a small library comprised of high-quality DMLs may better resolve the cellular landscape and avoid the inevitable noise that would result from a larger library comprised of lower quality DMLs; second, the design of custom methylation arrays with potential clinical use will be simplified using a small number of precise markers. Finally, having a smaller reference library would be more technically feasible and less costly for researchers, given that fewer CpGs would need to be profiled. Our simulation study results showed that even small improvements in prediction performance for RESET resulted in lower FDR compared to the Legacy approach for all differences in the underlying cellular composition when the sample size of each group was 500 (Figure 5A). Since EWAS typically entails statistical testing of hundreds of thousands to millions of CpGs, the potential consequence is that thousands of CpGs are incorrectly classified as differentially methylated when the Legacy versus RESET is used to construct reference libraries for deconvolution. Finally, in both data applications, cell proportion estimates obtained by using RESET resulted in an improved ability to explain variation in whole-blood-derived DNA methylation signatures. The consequence of this is improved statistical power for EWAS adjusted for cell-type composition when cell proportion estimates are obtained using the RESET versus the Legacy approach.

While our method resulted in improved accuracy of reference-based deconvolution estimates, lower FDR, and more variability in CpG-specific methylation explained in the context of EWAS adjusted for cell composition, RESET, and this study are not without limitations. The data sets used to build the reference libraries and benchmark the performance of our approach against the Legacy method only consisted of relative fractions of the six major leukocyte components of whole blood (e.g., CD4T, CD8T, B cells, NK cells, monocytes, and granulocytes), despite there being a myriad of other cell types and specific states of cells present in whole blood. In DNA methylation data, the main source of variability between isolated cell types is captured in the first two dimensions of the data. The Legacy approach exploits this fact using a fixed number of differentially methylated sites (usually 100 per cell-type) to capture that variability. However, as the cell lineages diverge, cell specialization drifts the epigenetic marks in a smaller proportion of CpGs. This translates into cell states/subtypes that are challenging to discern using surface/nuclear markers or even functional essays. As such, these states/subtypes fall closer to each other, rendering the assumption of a fixed number of sites untenable. Our algorithm makes no such assumption, and we hypothesize that our method will further outperform the Legacy approach in these situations. Additionally, while five independent data sets were used to evaluate and benchmark the performance of RESET, we note that data sets containing both DNA methylation data and gold-standard measurements of cell composition (e.g., flow cytometry) in the same set of samples are relatively rare, limiting the number of viable data sets to use in our comparisons. Further, only seven different fixed reference library sizes were considered in our evaluation ( $J^{*}=72,\;120,\;180,\;240,\;300,\;360,\;540$ ). While it is unclear what the performance of our approach would look like for larger library sizes, the results presented by Koestler et al. suggest diminishing returns on performance for libraries with more than 500 CpGs, motivating our decision to consider libraries with 540 or fewer CpGs (Koestler et al., 2016). A potential limitation of the Modified DSC is that this metric seems to favor smaller library sizes, specifically reference libraries with less than 100 loci. This could be due to the phenomenon known as the curse of high dimensionality, which is an occurrence when calculating Euclidean distances in high dimensional settings (Mirkes et al., 2020; Sarkar and Ghosh, 2020). In high dimensions, data can become noisy, so clusters of data can become more saturated, making the space between them (distances) harder to uncover. One possible way to overcome this would be to use the same algorithm but use a different distance metric (e.g., Manhattan distance) (Mirkes et al., 2020). It is also worth mentioning that the $R^{2}$ for NK cells using RESET was lower across all libraries compared to the Legacy approach; however, the lowest performance still had an $R^{2}$ of 0.78. The reasons for this lowered performance could be explained as the original Reinius dataset used for training the data had a known technical problem derived from potential cross-contamination of CD8T cells into their NK reference (Salas et al., 2018). As such, the reference cannot fully resolve between NK, CD4T, and CD8T cells and the difficulty that arises in distinguishing between these cell types. An additional limitation of RESET is its computational burden compared to other methods. This issue could be resolved by utilizing a lower-level programming language or the use of more parallelization of computations. Moreover, our algorithm also was only performed on whole-blood DNA methylation data; however, in principle, it can easily be extended to other solid tissue types where gold-standard measurements of the cell proportions are not as feasible to obtain. Finally, while this research utilized data from the Illumina HumanMethylation450 array, we note that this method is generalizable to other platforms (whole-genome bisulfite sequencing, Illumina HumanMethylationEPIC BeadArray, etc.).

This research was motivated by the need for accounting for cellular landscape when DNA methylation is analyzed in heterogeneous tissue, and further, by the need to remove requirements and arbitrary decisions other methods need in order to build reference libraries for the purpose of reference-based cell mixture deconvolution. Future work includes improving the accuracy of cell proportion estimates in the absence of a training data set by considering different distance metrics, building reference libraries for tissue types, and extending this algorithm to explore performance in samples with more than six cell types. The utilization of RESET will allow researchers to save money and remove arbitrary decisions in the reference library selection process while maintaining accuracy. RESET introduces a new way of thinking for the future of reference library selection in the absence of a training data set and is a useful approach for library construction for EWAS.

## Data Availability

The R scripts for the analyses presented here are available on GitHub at https://github.com/ShelbyBellGlenn/RESET_Code. The data sets used are all publicly available through GEO.

## Abbreviations

CMD, Cell Mixture Deconvolution; DML, Differentially Methylated Loci; DNAm, DNA methylation; DSC, Dispersion Separability Criterion; FDR, False Discovery Rate; EWAS, Epigenome Wide Association Studies; RESET, Reference Library Selection without a Training Dataset; RMSE, Root Mean Squared Error.
