# Supplementary material for: A Novel Framework for the Identification of Reference DNA Methylation Libraries for Reference-Based Deconvolution of Cellular Mixtures
Source: Front Bioinform. 2022 Mar 21;2:835591. doi: 10.3389/fbinf.2022.835591 (PMC9004796; doi:10.3389/fbinf.2022.835591)
Supplement: Supplementary file 1 [file Table1.DOCX]

**Algorithm for Simulation Study to Compare FDR**

1. Randomly sample *G* = 1,000 CpGs from the Illumina HumanMethylation450 array.
2. Estimate the dispersion parameter within the three combined testing sets for each of the *G* randomly selected CpGs, $\hat{\phi}_{g}, g=1,\ldots,G.$ Also estimate the cell-specific mean for each of the *G* CpGs, $m_{kg}, g=1,\ldots,G$ and *k* = 1,…,*K* using the Reference dataset [5].
3. Get random cell distributions for the two groups.
   1. For the first group, use a Dirichlet distribution to simulate the cell distribution, $\boldsymbol{w}^{(1)}.$ The concentration parameters are $\boldsymbol{\nu}^{(1)}=[\nu^{\left( 1 \right)},\nu^{\left( 1 \right)},\ldots,\nu^{\left( 1 \right)}]$.
   2. For the second group, use a Dirichlet distribution to simulate the cell distribution, $\boldsymbol{w}^{(2)}.$ The concentration parameters are $\boldsymbol{\nu}^{(2)}=[\nu^{\left( 2 \right)},\nu^{\left( 2 \right)},\ldots,\nu^{\left( 2 \right)}]$.
4. Simulate beta-values for each group, for each of the *G* CpGs using a beta-distribution.
   1. For the $n_{1}$ samples in group 1, we use mean $\boldsymbol{w}^{(1)}\boldsymbol{m}_{g}^{T}$ and variance $\frac{(1-\boldsymbol{w}^{(1)}\boldsymbol{m}_{g}^{T})\boldsymbol{w}^{(1)}\boldsymbol{m}_{g}^{T}}{1+ \hat{\phi}_{g}}$ to sample beta-values $Y_{ig}^{(1)}.$
   2. For the $n_{2}$ samples in group 2, we use mean $\boldsymbol{w}^{(2)}\boldsymbol{m}_{g}^{T}$ and variance $\frac{(1-\boldsymbol{w}^{(2)}\boldsymbol{m}_{g}^{T})\boldsymbol{w}^{(2)}\boldsymbol{m}_{g}^{T}}{1+ \hat{\phi}_{g}}$ to sample beta-values $Y_{ig}^{(2)}.$
5. Randomly sample cell type predictions for each sample (${\hat{\boldsymbol{w}}}_{il}^{(1)} and {\hat{\boldsymbol{w}}}_{il}^{(2)}$). To do this, use the cell-specific uncertainty estimates, $\hat{\tau}_{kl}^{2}$. More specifically, we sample $n_{1}$ for group 1 by sampling from a multivariate normal distribution with mean $\boldsymbol{w}^{(1)}$ and variance-covariance, $\Sigma_{l}^{(1)}=diag\left( \hat{\tau}_{kl}^{2} \right), k=1,2,\ldots,K$ and *l* is either the Legacy approach or the Modified DSC approach. This gives us the estimate ${\hat{\boldsymbol{w}}}_{il}^{(1)}$. We similarly get an estimate for ${\hat{\boldsymbol{w}}}_{il}^{(2)}$ by using $\boldsymbol{w}^{(2)}$ and $\Sigma_{l}^{(2)}=diag\left( \hat{\tau}_{kl}^{2} \right), k=1,2,\ldots,K$.
6. Fit the above regression model to each of the *G* CpGs, adjusting for cell composition using the cell proportion predictions obtained in step 4. Use the model fit to test the hypothesis $H_{0} : \alpha_{1g}=0, for g=1,2,\ldots,G.$
7. Get the FDR for each method assuming a nominal cutoff of 0.05 for significance.
8. Repeat steps 1-7.
