# Supplementary material for: A Novel Framework for the Identification of Reference DNA Methylation Libraries for Reference-Based Deconvolution of Cellular Mixtures
Source: Front Bioinform. 2022 Mar 21;2:835591. doi: 10.3389/fbinf.2022.835591 (PMC9004796; doi:10.3389/fbinf.2022.835591)

**Supplementary Figure 1: Data Application Results for the DML Reference Library of Size 72. (A)** Scatter plots of the predicted cell type proportions using the Legacy library of 72 CpGs (x-axis) and the RESET library of 72 CpGs (y-axis) (left panel) as well as the distribution of the difference in $R^{2}$ obtained from the RESET and Legacy libraries of size 72 CpGs (right panel) applied to the Liu data set. **(B)** Scatter plots of the predicted cell type proportions using the Legacy library of 72 CpGs (x-axis) and the RESET library of 72 CpGs (y-axis) (left panel) as well as the distribution of the difference in $R^{2}$ obtained from the RESET and Legacy libraries of size 72 CpGs (right panel) applied to the Hannum data set.


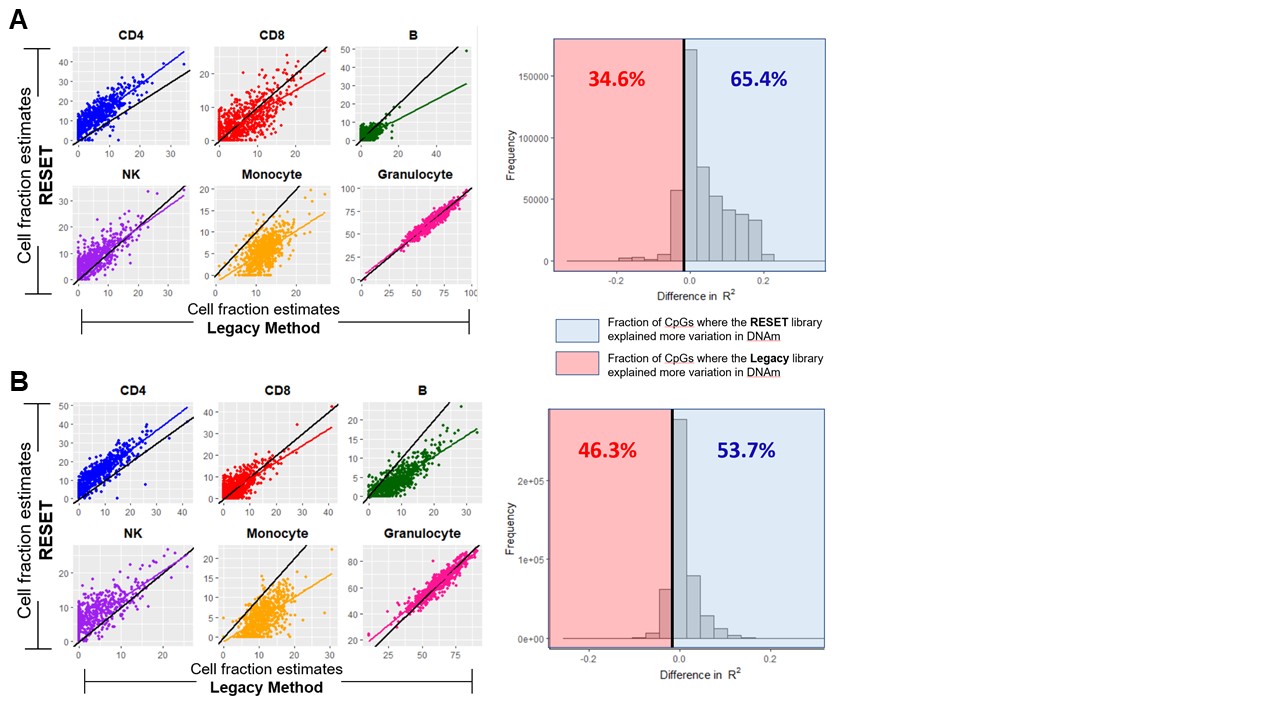


**Supplementary Figure 2: Data Application Results for the DML Reference Library of Size 180. (A)** Scatter plots of the predicted cell type proportions using the Legacy library of 180 CpGs (x-axis) and the RESET library of 180 CpGs (y-axis) (left panel) as well as the distribution of the difference in $R^{2}$ obtained from the RESET and Legacy libraries of size 180 CpGs (right panel) applied to the Liu data set. **(B)** Scatter plots of the predicted cell type proportions using the Legacy library of 180 CpGs (x-axis) and the RESET library of 180 CpGs (y-axis) (left panel) as well as the distribution of the difference in $R^{2}$ obtained from the RESET and Legacy libraries of size 180 CpGs (right panel) applied to the Hannum data set.


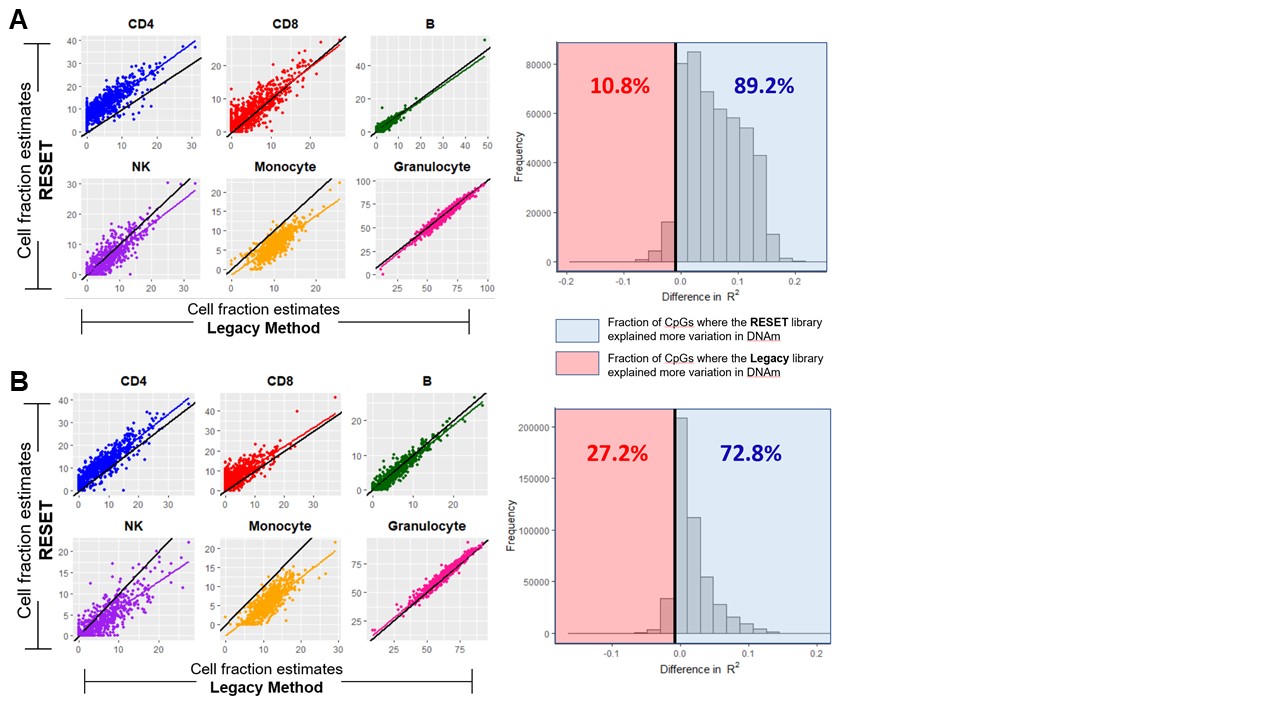


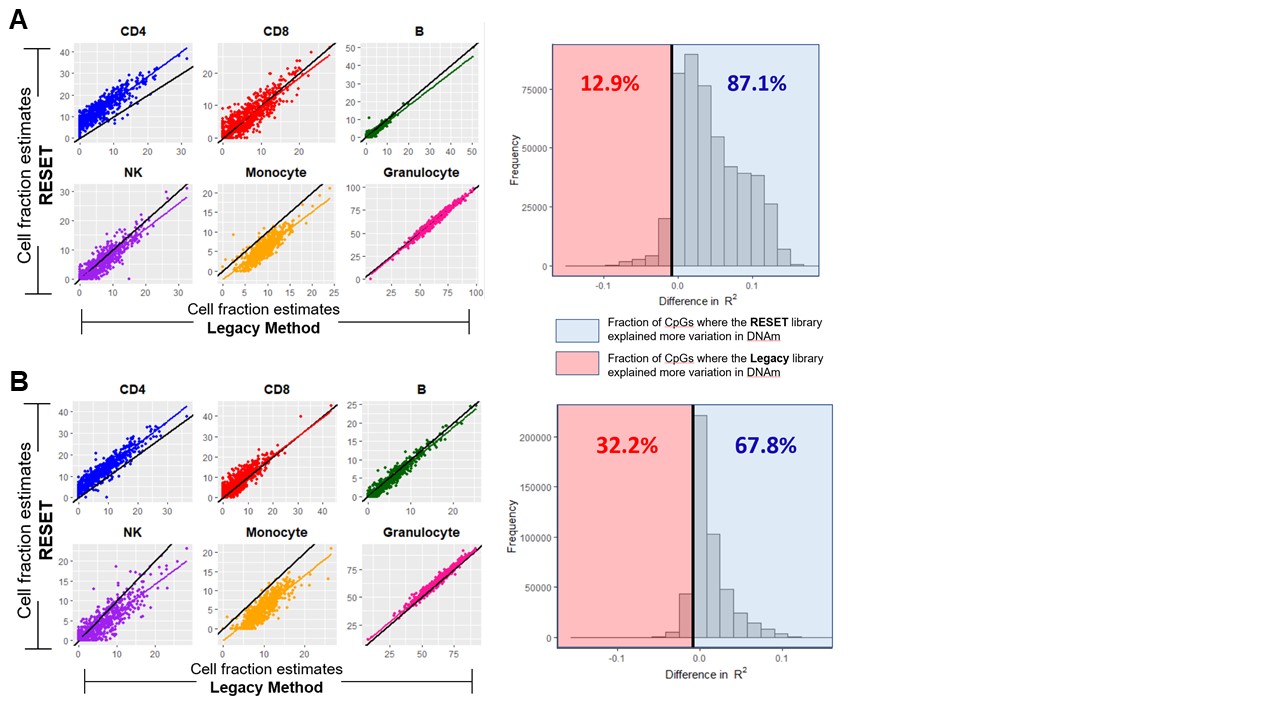
**Supplementary Figure 3: Data Application Results for the DML Reference Library of Size 240. (A)** Scatter plots of the predicted cell type proportions using the Legacy library of 240 CpGs (x-axis) and the RESET library of 240 CpGs (y-axis) (left panel) as well as the distribution of the difference in $R^{2}$ obtained from the RESET and Legacy libraries of size 240 CpGs (right panel) applied to the Liu data set. **(B)** Scatter plots of the predicted cell type proportions using the Legacy library of 240 CpGs (x-axis) and the RESET library of 240 CpGs (y-axis) (left panel) as well as the distribution of the difference in $R^{2}$ obtained from the RESET and Legacy libraries of size 240 CpGs (right panel) applied to the Hannum data set.


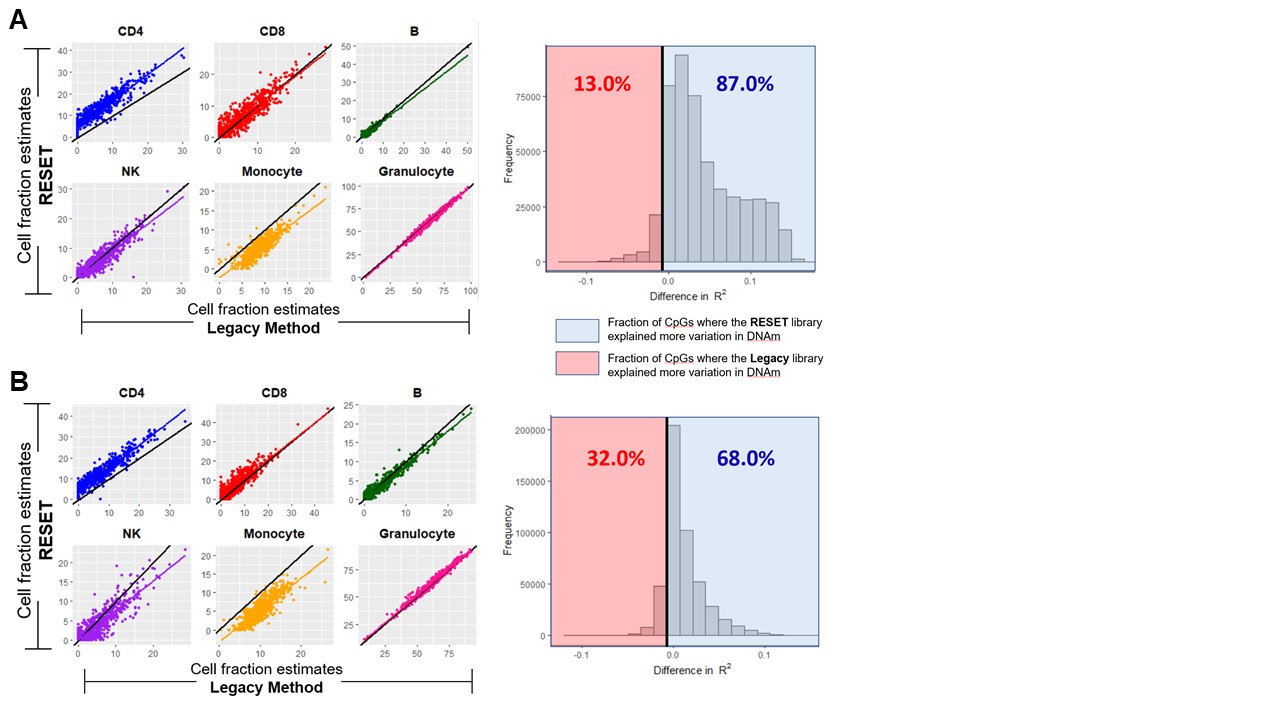
**Supplementary Figure 4: Data Application Results for the DML Reference Library of Size 300. (A)** Scatter plots of the predicted cell type proportions using the Legacy library of 300 CpGs (x-axis) and the RESET library of 300 CpGs (y-axis) (left panel) as well as the distribution of the difference in $R^{2}$ obtained from the RESET and Legacy libraries of size 300 CpGs (right panel) applied to the Liu data set. **(B)** Scatter plots of the predicted cell type proportions using the Legacy library of 300 CpGs (x-axis) and the RESET library of 300 CpGs (y-axis) (left panel) as well as the distribution of the difference in $R^{2}$ obtained from the RESET and Legacy libraries of size 300 CpGs (right panel) applied to the Hannum data set.

**Supplementary Figure 5: Data Application Results for the DML Reference Library of Size 360. (A)** Scatter plots of the predicted cell type proportions using the Legacy library of 360 CpGs (x-axis) and the RESET library of 360 CpGs (y-axis) (left panel) as well as the distribution of the difference in $R^{2}$ obtained from the RESET and Legacy libraries of size 360 CpGs (right panel) applied to the Liu data set. **(B)** Scatter plots of the predicted cell type proportions using the Legacy library of 360 CpGs (x-axis) and the RESET library of 360 CpGs (y-axis) (left panel) as well as the distribution of the difference in $R^{2}$ obtained from the RESET and Legacy libraries of size 360 CpGs (right panel) applied to the Hannum data set.


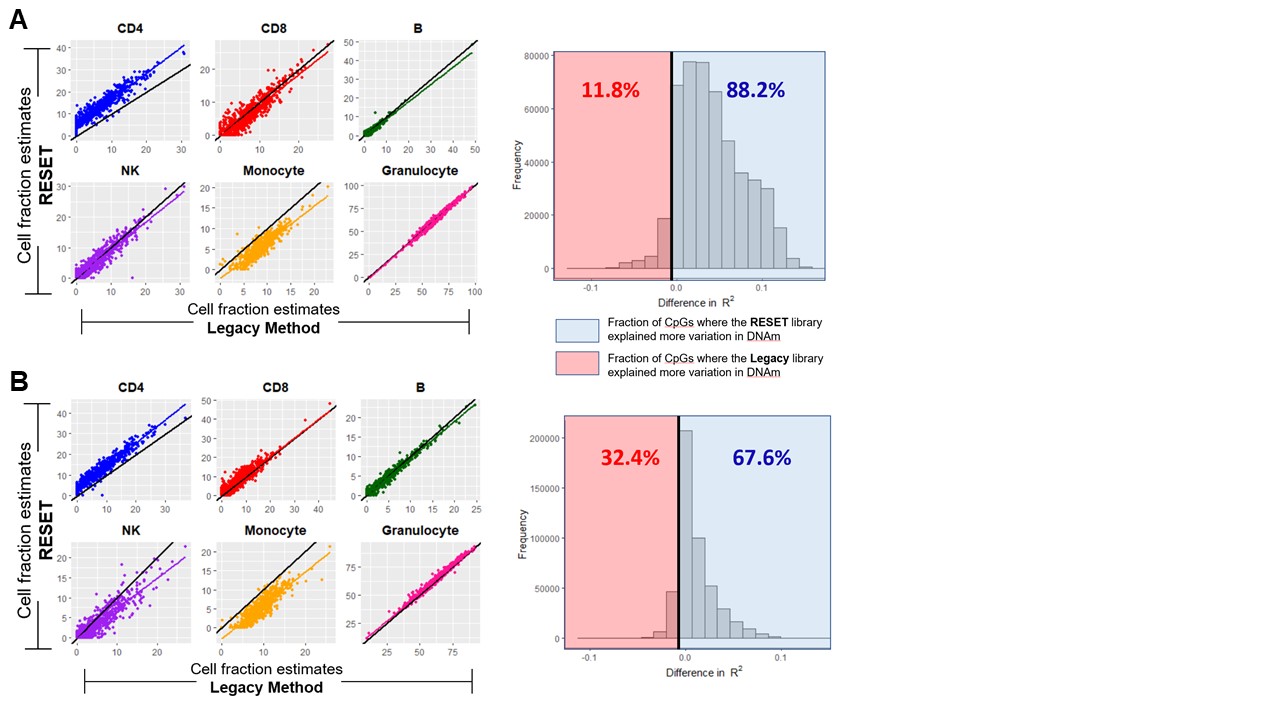


**Supplementary Figure 6: Data Application Results for the DML Reference Library of Size 540. (A)** Scatter plots of the predicted cell type proportions using the Legacy library of 540 CpGs (x-axis) and the RESET library of 540 CpGs (y-axis) (left panel) as well as the distribution of the difference in $R^{2}$ obtained from the RESET and Legacy libraries of size 540 CpGs (right panel) applied to the Liu data set. **(B)** Scatter plots of the predicted cell type proportions using the Legacy library of 540 CpGs (x-axis) and the RESET library of 540 CpGs (y-axis) (left panel) as well as the distribution of the difference in $R^{2}$ obtained from the RESET and Legacy libraries of size 540 CpGs (right panel) applied to the Hannum data set.


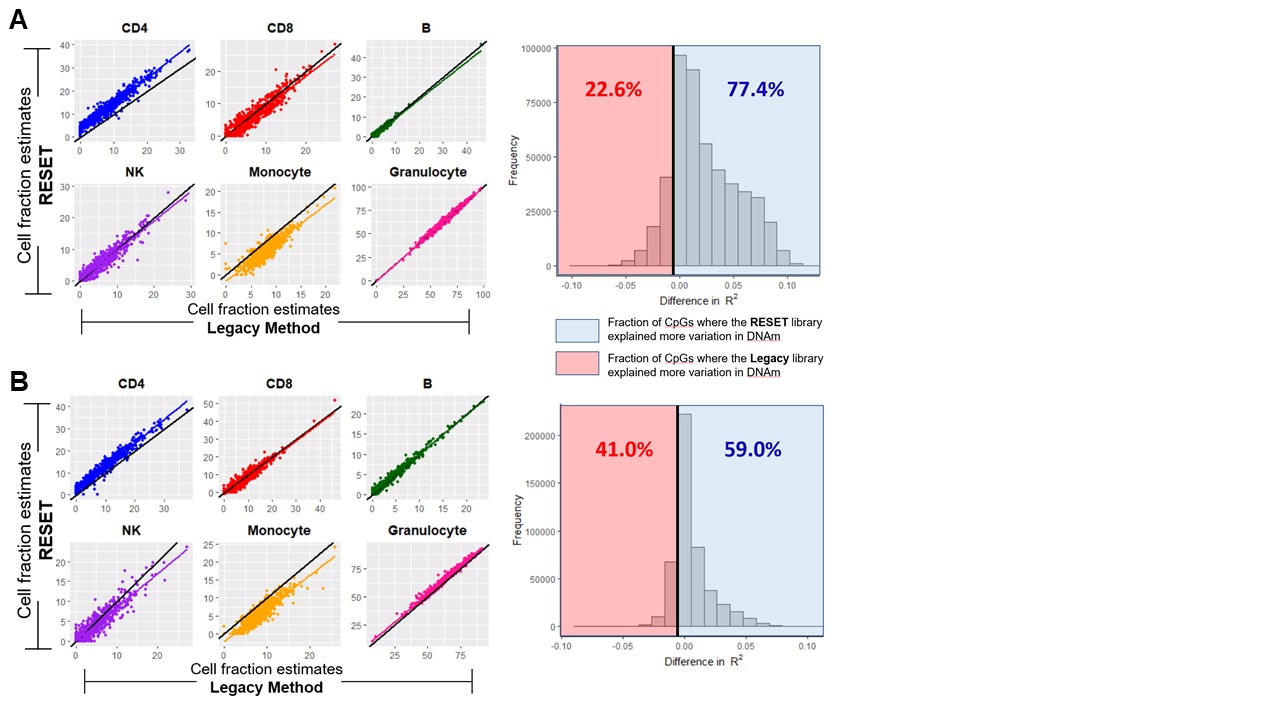

Supplement: Supplementary file 2 [file Table2.DOCX]
